# Comparison of sleep microarchitecture in screen failure subjects with insomnia complaints and randomized subjects from two phase 3 studies on insomnia disorder

**DOI:** 10.1192/j.eurpsy.2023.612

**Published:** 2023-07-19

**Authors:** T. Di Marco, Y. Dauvilliers, T. Scammell, I. Djonlagic, A. Datta, G. Zammit, D. Seboek Kinter, N. Tjiptarto, J. Donoghue

**Affiliations:** ^1^Idorsia Pharmaceuticals Ltd, Allschwil, Switzerland; ^2^Centre Hospitalier Universitaire de Montpellier, Montpelier, France; ^3^Beth Israel Deaconess Medical Center, Boston, United States; ^4^University Children’s Hospital Basel, Basel, Switzerland; ^5^Clinilabs Drug Development Corporation, New York; ^6^Beacon Biosignals, Inc., Boston, United States

## Abstract

**Introduction:**

Daridorexant, a dual orexin receptor antagonist, was shown to be effective and safe in improving sleep and daytime functioning in subjects with insomnia in two Phase 3 studies. All patients screened had subjective insomnia, but many did not meet the study eligibility criteria.

**Objectives:**

As the randomized subjects represent only a subsection of the real-world population, we analyzed differences in sleep microarchitecture between included and excluded subjects in both studies.

**Methods:**

Out of 7016 screened subjects, 1851 randomized (included) subjects and 602 screen-failure (SF, excluded) subjects that had at least 1 scored eligibility polysomnography available, were included in this analysis. For the remaining SF subjects, no scored polysomnography was available. The randomized subjects met the DSM 5 insomnia disorder criteria and objective and subjective criteria for disrupted sleep. The main reasons for the 602 SF subjects were not meeting at least 1 objective sleep criteria for sleep onset latency, sleep maintenance, or total sleep time, however all excluded subjects had subjective insomnia. Delta (1-3Hz), theta (4-7Hz), alpha (8-12Hz), and beta (13-38Hz), band spectral power of sleep EEGs were estimated using multi-taper spectrograms (2s window/1s overlap). Relative power was computed using the sum of these four band powers within the 2s window as the denominator. The resulting relative and band power ratios were then aggregated to 30s epochs and assessed by sleep stages (N1, N2, N3, REM, Awake). Sleep spindles (amplitude, peak frequency, oscillation count, symmetry index, slow oscillation phase peak, duration, density, and dispersion) were calculated in N2 sleep using an open-source Luna package. Statistical analysis was done using a univariate analysis of spindle and spectral features via linear mixed-effects regression.

**Results:**

Age and sex distribution were similar between groups (Median age: 59vs59 years and 68%vs70% Females for included and excluded subjects, respectively). Included subjects had higher relative alpha power (5.6%; p<0.001) and lower relative delta power (-2.3%; p0.031) in N1 than excluded subjects. The mean relative spectral power did not differ significantly for other relative powers in N1 and for any relative powers in stages N2, N3, REM and AWAKE. Included subjects had lower spindle density (-9.8%; p0.005) than excluded subjects. Other spindle features did not significantly differ between the groups.

**Conclusions:**

This comparison of sleep architecture between included and excluded subjects, showed only minor differences in sleep microarchitecture. This suggests that the sleep microarchitecture of the randomized subjects is similar to a broader insomnia population that has subjective insomnia but that does not meet all eligibility criteria including objective and subjective sleep duration thresholds.

**Disclosure of Interest:**

T. Di Marco Employee of: Idorsia Pharmaceuticals Ltd, Y. Dauvilliers Consultant of: Idorsia Pharmaceuticals Ltd, T. Scammell Consultant of: Idorsia Pharmaceuticals Ltd, Neurocrine, Epilog, Roche and Jazz Pharmaceuticals, I. Djonlagic Grant / Research support from: NIH, Consultant of: Idorsia Pharmaceuticals Ltd, UCSD, A. Datta Consultant of: Idorsia Pharmaceuticals Ltd, Neurocrine, Epilog, Roche and Jazz Pharmaceuticals, G. Zammit Grant / Research support from: Idorsia Pharmaceuticals Ltd, Consultant of: Idorsia Pharmaceuticals Ltd, D. Seboek Kinter Employee of: Idorsia Pharmaceuticals Ltd, N. Tjiptarto Grant / Research support from: Idorsia Pharmaceuticals Ltd, J. Donoghue Grant / Research support from: Idorsia Pharmaceuticals Ltd.

